# Metabolic and Community Synergy of Oral Bacteria in Colorectal Cancer

**DOI:** 10.1128/mSphere.00102-16

**Published:** 2016-05-11

**Authors:** Kaitlin J. Flynn, Nielson T. Baxter, Patrick D. Schloss

**Affiliations:** Department of Microbiology and Immunology, University of Michigan Medical School, Ann Arbor, Michigan, USA; University of Wisconsin

**Keywords:** colorectal cancer, microbial ecology, microbiome, oral microbiology, pathogenesis

## Abstract

The oral periodontopathic bacterium *Fusobacterium nucleatum* has been repeatedly associated with colorectal tumors. Molecular analysis has identified specific virulence factors that promote tumorigenesis in the colon. However, other oral community members, such as members of the *Porphyromonas* spp., are also found with *F. nucleatum* on colonic tumors, and thus, narrow studies of individual pathogens do not take community-wide virulence properties into account. A broader view of oral bacterial physiology and pathogenesis identifies two factors that could promote colonization and persistence of oral bacterial communities in the colon. The polymicrobial nature of oral biofilms and the asaccharolytic metabolism of many of these species make them well suited to life in the microenvironment of colonic lesions. Consideration of these two factors offers a novel perspective on the role of oral microbiota in the initiation, development, and treatment of colorectal cancer.

## INTRODUCTION

The gut microbiota is known to potentiate tumorigenesis in colorectal cancer (CRC) (1). Strains of common members in the gut microbiota such as *Bacteroides fragilis*, *Enterococcus faecalis*, and *Escherichia coli* are frequently associated with CRC tumors, but molecular studies reveal only weak pathogenic features of these bacteria when studied in isolation (2). In contrast, *Fusobacterium nucleatum* is regularly identified in studies of CRC tumor microbiota, often as a consortium with other oral microbes (3). In particular, *Porphyromonas* spp. (often *P. asaccharolytica*) are among the most consistently enriched taxa in CRC patients. Interestingly, *F. nucleatum* and *Porphyromonas gingivalis* are known to synergistically promote oral cancer progression (4). Studies of these two periodontal pathogens have identified several virulence mechanisms that promote both bacterial survival and carcinogenesis (5). The specific virulence properties have been well reviewed elsewhere and include the abilities to invade the gut submucosa and epithelium, disrupt oncogene signaling, disrupt cell-cell adhesion, promote inflammation, and inhibit natural killer and cytotoxic T cells, promoting tumor proliferation and progression (2, 5).

In addition to *Fusobacterium* and *Porphyromonas*, several other oral commensal and periodontopathic bacteria are consistently enriched in the cancerous colon. These bacteria, including members of the *Peptostreptococcus*, *Prevotella*, *Parvimonas*, and *Gemella* genera have been repeatedly shown to be effective biomarkers for detecting CRC, yet none have been studied individually to determine species-specific virulence factors that allow for colon colonization (6–8). The consistent cooccurrence of these oral species suggests that there are community-wide properties that promote persistence or tumorigenesis. Two opportunistic factors that play a role in periodontal pathogenesis but have not yet been appreciated with respect to oral bacteria in CRC are the propensity of the bacteria to form biofilms and their anaerobic asaccharolytic metabolism. By building from what is known about the synergistic activities of these bacteria in periodontal disease and oral cancer, we can better understand the mechanisms by which the microbiota modulate colon tumorigenesis and design specific interventions for this subtype of CRC.

## POLYMICROBIAL COMMUNITIES IN PERIODONTAL AND COLORECTAL DISEASE

Periodontitis is an inflammatory disease of the tissue surrounding teeth and is the major cause of tooth loss worldwide (9). The disease occurs following a dramatic shift in the oral microbial community resulting in openings in the niche space that is filled by pathogens (9). Periodontal biofilms form in three stages (10). Early colonizers of the periodontal biofilm include *Streptococcus* spp. and *Actinomyces* spp., which bind to teeth and act as a bridging organism for binding early and intermediate colonizers such as *F. nucleatum* (10). These species express multiple adhesins and can aggregate with both early and late colonizers like *P. gingivalis*. A “driver-passenger” model has been proposed to describe the final stage of periodontal biofilm formation (9). After the healthy community is disrupted, “driver” pathogenic organisms such as *P. gingivalis* can produce virulence factors that continue to transform the biofilm community structure, affect bacterial load and gene expression of “passenger” species, and subvert the host immune response (9, 11). The polymicrobial synergy and dysbiosis (PSD) model expands on the “driver-passenger” model by postulating that temporally coordinated gene and species combinations foster a periodontal disease state (12). Specifically, this model postulates that intermediate “accessory” species such as *F. nucleatum* can express multiple adhesins, proteolytic enzymes, and proinflammatory surface ligands that invade oral epithelial cells and sustain an environment that enhances the virulence of later-arriving “keystone” species such as *P. gingivalis* in the periodontic biofilm (12). The resulting periodontopathic community supports itself nutritionally and increases the occurrence of proinflammatory tissue destruction (12).

Cooperation between oral species likely equips them well for a life in the colon. As periodontal species are adept at coaggregation using adhesins in the anaerobic subgingival space, colon environment conditions could facilitate these interactions as well. Indeed, biofilms containing oral microbes have been isolated from both healthy colonic mucosa and CRC tumors (13). Many CRC and adenoma biofilm samples contained a higher abundance of invasive *F. nucleatum* as detected by fluorescent *in situ* hybridization (FISH) and 16S rRNA gene sequencing (13), consistent with previous microbiome-based analyses. Importantly, all colon biofilms isolated in this study were polymicrobial, and *F. nucleatum* was not isolated from healthy colon biofilms. Furthermore, all biofilm-positive tumor samples and a subset of matched biofilm-positive normal tissue samples contained bacteria that had invaded the tumor cells as detected by FISH (13). In this way, cancer-associated biofilms found in the colonic mucosa are similar in both community membership and invasiveness to those of a periodontopathics state in the subgingival space.

Similar to the PSD model in the oral environment, a “driver-passenger” model has been proposed as a mechanism for polymicrobial interactions in CRC (2, 14). In this model, “driver” bacteria promote epithelial DNA damage and initial tumorigenesis, creating an altered niche in the colon for colonization by “passenger” bacteria (14). Indeed, it is known that the bacteria present on developing CRC tumors differ temporally (14). 16S rRNA gene sequencing results suggest that driver and passenger species maintain a complex relationship, possibly in a bacterial biofilm, as drivers such as *F. nucleatum* and *Porphyromonas* spp. remain on the tumor in a polymicrobial community (13). It is important to note that the colon “driver-passenger” model was proposed before the discovery of multiple oral species found on colon tumors, and thus, it was initially proposed that *F. nucleatum* was merely a “passenger” species (14). In light of what we now know about *F. nucleatum* activity in the colon, this bacterium may be better described as a “driver” species in this model. Specifically, molecular studies of *F. nucleatum* have discovered the virulence protein FadA and its role in transformation of epithelial cells and promotion of colon tumorigenesis (15). Furthermore, *F. nucleatum* potentiates colon tumorigenesis in the *Apc*^Min/+^ mouse model of colorectal cancer, as daily administration of *F. nucleatum* increased both colon tumor number and tumor-infiltrating myeloid cell recruitment (16). Thus, as in the oral cavity, *F. nucleatum* appears to be a sufficient “driver” species that initiates tumorigenesis. Further studies will elucidate whether the presence of *F. nucleatum* promotes colonization of “passenger” oral species that thrive in the inflammatory environment created in the colon.

Studies of the CRC microbial community reveal interesting associations between species and pathology. Deep sequencing studies of CRC and matched normal tissues reveal a cooccurrence of oral species and oral anaerobes such as *F. nucleatum*, *Leptotrichia*, and *Campylobacter* (17). Pairwise correlation of these microbial sequence counts revealed that these three species exist in a cooccurrence network along with *Porphyromonas* in CRC tissue (17). Furthermore, isolation of the *Campylobacter* species from these samples show that it is able to coaggregate with *F. nucleatum* (17). This finding supports the hypothesis that *F. nucleatum* facilitates colon tissue infection by acting as a vector for oral microbes with compatible adhesins (17). Polymicrobial interactions likely have a role in carcinogenesis, as mouse studies using *F. nucleatum* alone require the administration of bacteria daily for 8 weeks to establish tumors, implying that *F. nucleatum* cannot stably colonize the gut on its own (16). Thus, our current models for directly testing the contribution of one or many oral species are insufficient. One interesting approach to assessing oral microbe activity in the colon would be to perform metatranscriptomic analysis on bacteria isolated from human CRC samples. This type of study could probe for biofilm matrix proteins, adhesins, quorum-sensing molecules, and other virulence proteins that may promote a stabilized oral community in the colon. Once a core set of microbes or functions is elucidated, perhaps a mouse model of infection would be more achievable and allow for direct testing of antibiofilm therapeutics or probiotic interventions.

## ASACCHAROLYTIC METABOLISM OF ORAL PATHOGENS IN THE COLON ENVIRONMENT

A hallmark of periodontitis is a microbial community shift from protective facultative anaerobes to strict anaerobic species (9). These species are typically asaccharolytic and proteolytic, that is, they do not use sugar or carbon fermentation for energy but digest peptides and amino acids for carbon and nitrogen (18). Subgingival species metabolize nitrogenous compounds from gingival crevicular fluid (GCF) (18). This metabolism creates a neutral pH, releases short-chain fatty acids (SCFAs) and ammonia into the subgingival space. The environmental disruption further stimulates GCF efflux and supports the proteolytic activity of *Prevotella intermedia* and other species, which continue to facilitate a neutral pH environment, supporting the colonization of pathogenic organisms like *P. gingivalis* (18). Once established, a microbial consortium of *F. nucleatum*, *Eubacterium*, *Campylobacter*, *P*. *intermedia*, and *P. gingivalis* use membrane-bound or secreted proteases to continue to feed off of proteins in GCF and desquamated epithelial cells. This metabolic synergy promotes a nutrient-rich disease state that supports growth of a diverse and cooperative microbial ecosystem.

Remarkably, while each of these species uses the same nutrient source, their metabolic pathways differ. Bacterial preference for different nutrients reduces competition in the oral biofilm. Indeed, *P. gingivalis* preferentially degrades dipeptides, while *F. nucleatum* and *P. intermedia* prefer amino acids to larger molecules (18). Furthermore, proteolytic species like *P. gingivalis* can provide amino acids to nonproteolytic *F. nucleatum*. In this way, *F. nucleatum* and *P. intermedia* create a neutral pH environment that promotes colonization of *P. gingivalis*, which then provides amino acids as carbon and nitrogen sources. Continued breakdown of host proteins stimulates an immune response that then provides more nutrients for the biofilm to subsist on (9). Meanwhile, bacterial proteases inhibit complement-directed killing by the immune system (3). This coordinated metabolism was crucial for the development of periodontitis as transcriptomic studies of periodontal tissues show a highly conserved metabolic profile, despite a diverse microbial composition between patients (11). The authors of this study speculated that the genetic capacity of the disease state matters more to pathogenesis that the specific species present. That is, many organisms can fill conserved metabolic niches, and thus, the metabolic properties facilitating a pathogenic community are more important than the virulence properties of one species or another species alone.

A similar synergistic effect may occur when oral microbes inhabit the colon. The mucosa of the colon is anaerobic and more neutral in pH than the mouth or stomach (19). Likewise, gut mucosa and epithelium are constantly being sloughed off, providing nutrients and binding sites for adhesive bacteria (19, 20). Once the mucous layer is penetrated, invasive *F. nucleatum* can disrupt epithelial junctions by binding to E-cadherin (15, 20). The altered mucosal environment could then support the growth of other asaccharolytic anaerobes like *Porphyromonas* and *Peptostreptococcus*. Degradation of host proteins in the mouth and gut trigger an inflammatory response that ultimately provides more peptides for nutrient degradation by gut and oral species. This chronic inflammatory state would promote the development of CRC (21). Indeed, it has been shown that *F. nucleatum* facilitates a proinflammatory state in the CRC tumor microenvironment (16) and that these changes precipitate tumor formation. Thus, oral species can wreak havoc on the gut using the same virulence strategies that promote survival and persistence in the oral cavity.

A polymicrobial oral community can further damage the colon by the production of metabolites such as polyamines (22) and genotoxic reactive oxygen species (ammonia, hydrogen sulfide), which both protect the microbiota present and promote tumorigenesis (18). Metabolomic studies of colon biofilms have revealed an upregulation of polyamine metabolites spermine and diacetylspermine that cause DNA damage in colon tissues (23). This finding was specific to colon biofilms, as treatment with antibiotics reduced both the biofilm and diacetylspermine levels (23). Furthermore, polyamines are known to be essential for biofilm formation (24) and promote cancer cell proliferation. In this way, polymicrobial oral communities produce metabolites that disrupt healthy colonic metabolism, promoting tumor cell proliferation while protecting the stability of their biofilm (22, 23).

As mentioned above, oral asaccharolytic bacteria metabolize peptides and amino acids through three divergent pathways (18). These processes produce SCFAs that accumulate at high concentrations locally in the gingival crevice and are proinflammatory, prolonging disease in the mouth (18). However, in the gut, these same SCFAs produced from the fermentation of resistant starch by the gut microbiota have anti-inflammatory and proapoptotic effects, protecting the colon epithelium from the development of CRC (21). Thus, it is not clear how production of SCFAs by oral pathogens in the gut might influence tumorigenesis. It is possible that the anti-inflammatory activity of SCFAs in the colon may allow for immune subversion by oral pathogens. Metabolomic studies probing for SCFA concentrations on CRC tumors or biofilms have not been performed, but careful study of these samples would illuminate the effect of oral microbiota-produced SCFAs on tumor pathology.

## CONCLUSION

Given the recent advances in microbiome, metagenome, metatranscriptome, and metabolome analyses, we are learning how oral microbes contribute to colorectal tumorigenesis. An integrated view of the entire community found on CRC tumors suggests that tumorigenesis depends more on the structure and metabolic activities of a community of bacteria rather than just one specific pathogen. Thus, it is not just specific bacterial virulence factors nor disruption in epithelial signaling or promotion of inflammation that leads to CRC tumors, but all of these components in concert with a synergistic polymicrobial oral biofilm that creates an ideal tumor microenvironment for persistence and destruction.

The precise mechanism of dissemination from the oral cavity to the colon remains to be elucidated. Two routes have been proposed. The first route is through systemic dissemination via ulcerated gingival pockets that allow *P. gingivalis* and *F. nucleatum* to access the bloodstream (9). *F. nucleatum* has been implicated in a number of other diseases, including adverse pregnancy outcomes such as stillbirth, liver cirrhosis, and rheumatoid arthritis (9). Access to these extraoral sites is likely facilitated by bacteremic transfer. The second possible route of infection is via swallowing of oral bacteria that alter the state of the colon microbiota. However, because of the relatively low overall occurrence of the organisms in the colons of healthy individuals and the need for daily gavaging of *F. nucleatum* to establish tumors in mice, additional factors must promote colonization if oral inoculation is the main route of infection. Indeed, quantitative PCR (qPCR) analysis of tissue samples from 1,069 CRC patients found that only 13% contained *F. nucleatum* DNA (25). Thus, for colonization in this subset of patients, an already inflamed colon or perturbed community may precede or facilitate colonization by oral microbes. The precise factors promoting colonization require further study, as they may be of therapeutic interest.

**FIG 1:**
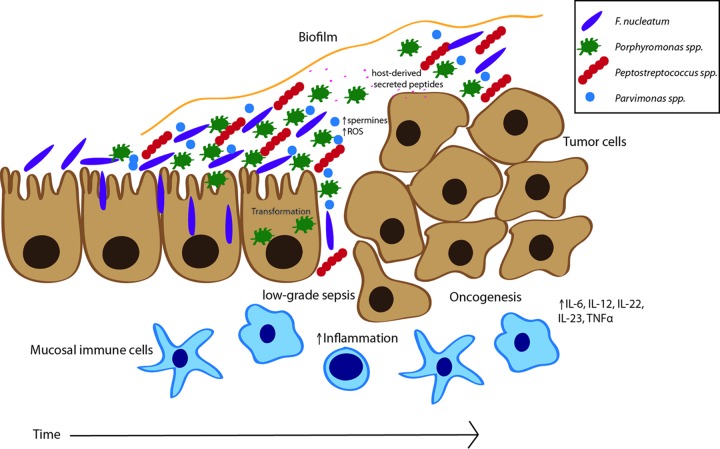
A model of oral microbial activities in colon tumorigenesis. In this model, oral microbes such as *F. nucleatum* colonize the gut epithelial surface. *F. nucleatum* may act as a bridging organism, allowing for other oral microbes to bind via compatible adhesins (*Porphyromonas* spp., *Peptostreptococcus* spp., and *Parvimonas* spp.). *F. nucleatum* and *Porphyromonas* can invade epithelial cells, disrupting signaling and promoting transformation. The oral microbes form a biofilm community that alters epithelial tight junctions and promotes infiltration and inflammation from mucosal immune cells. Transformation of epithelial cells leads to an oncogenic synergy where host-secreted peptides feed asaccharolytic oral microbes, who in turn produce reactive oxygen species (ROS) and polyspermines, promoting both biofilm formation and continued inflammatory responses as the tumor grows. IL-6, interleukin 6; TNFα, tumor necrosis factor alpha.

In light of recent studies, we propose a model of polymicrobial synergy that leads to oral-microbe-induced colon tumorigenesis (Fig. 1). First, changes in the gut microbiota allow oral bacteria to colonize the gut mucosa. Invasive bacteria such as *F. nucleatum* can disrupt the epithelial barrier, while also functioning as a bridging organism to link early and late-colonizing microbes together. These oral microbes may modify the pH by production of ammonia and hydrogen sulfide, as well as promote inflammation. Subsequent changes in the microenvironment promote the growth and biofilm formation of other oral species such as *Porphyromonas*, *Peptostreptococcus*, *Parvimonas*, and *Gemella*. In concert with *F. nucleatum*, these pathogens produce virulence factors that disrupt epithelial cell signaling and tight junctions, altering the permeability of the gut and promoting transformation and oncogenesis. As tumor tissues grow, peptides and proteins that are preferentially metabolized by the asaccharolytic oral microbes are released. A persistent state of inflammation continues to sustain the biofilm and to promote tumorigenesis in the colon. Thus, the power of the entire microbial tumor community, and not just known pathogens like *F. nucleatum*, must be appreciated in the design of mechanistic studies, microbiota-based screening tests, and therapeutic interventions targeting the activities of the microbial consortia for CRC.
